# Positron emission tomography in the diagnostic pathway for intracystic infection in adpkd and "cystic" kidneys. a case series

**DOI:** 10.1186/1471-2369-12-48

**Published:** 2011-09-29

**Authors:** Giorgina B Piccoli, Vincenzo Arena, Valentina Consiglio, Maria Chiara Deagostini, Ettore Pelosi, Anastasios Douroukas, Daniele Penna, Giancarlo Cortese

**Affiliations:** 1 Department of Clinical and Biological Sciences of the University of Turin, San Luigi Gonzaga Hospital, regione Gonzole 10, Orbassano (TO), 10043 Italy; 2 Deapartment of Radiology, Ospedale Maria Vittoria, via Cibrario 72, 10100 Torino, Italy; 3 IRMET SpA, Centro PET, via Onorato Vigliani 89, Torino, 10100 Italy

**Keywords:** Positron emission tomography, polycystic kidney disease, infection, kidney cysts, long-term antibiotic therapy

## Abstract

**Background:**

Intracystic infection, in Autosomal Dominant Polycystic Kidney Disease (ADPKD) and in kidneys with multiple cysts, is a diagnostic and therapeutic challenge, as conventional imaging techniques may not discriminate among "complicated" cysts (infection, bleeding, neoplasia), and as the clinical picture may be attenuated, in particular in early phases. Positron Emission Tomography with fluorodeoxyglucose (FDG-PET) was recently suggested as a tool to detect infection in ADPKD, in single cases and small series.

The aim of the study was to report on the role of FDG-PET in the work-up of 10 cases of suspected cystic infections, affected by ADPKD or with multiple kidney cysts.

**Methods:**

Observational study. Review of clinical charts and of the imaging data since the use of FDG-PET for detecting cystic infections (2008-2010).

**Results:**

In 2008-2010, 6 patients with ADPKD and 4 with multiple kidney cysts were referred for suspected intracystic infections (3 males, 7 females, aged 55-83 years, in all CKD stages); in one case the imaging was done in the work-up of a complicated "uremic" cyst. The clinical picture, the usual inflammatory markers and/or the conventional imaging techniques did not allow conclusive diagnosis at referral or during follow-up (ultrasounds in all, CT in 8/10). Nine patients displayed inflammatory signs (increase in C-reactive protein and other biochemical markers) and constitutional symptoms (fever in 9/10).

FDG-PET was positive in 6 cases (5 kidney and 1 liver cyst), was repeated during follow-up in 4 patients and was negative in 4 cases. In the positive cases, FDG-PET guided the therapeutic choices; in particular, the duration of therapy was supported by imaging data in the 4 cases with multiple scans. No relapse was recorded after discontinuation of antibiotic therapy in the treated patients. The negative cases did not develop clinical signs of cystic infection over follow-up.

**Conclusion:**

In this case series, the largest prospective one so far published and the only one including different types of renal cysts, FDG-PET is confirmed as a promising diagnostic tool for detecting intracystic infection in ADPKD and in multiple kidney cysts, and a potential guide for tailoring therapy. Further larger and multicenter studies are needed to evaluate the cost-benefit ratio and the limits of this imaging technique in the clinical setting.

## Background

Autosomal Dominant Polycystic Kidney Disease (ADPKD) is the most common monogenic severe kidney disease, with an average incidence of 1 in 800 live births [1,2]. In spite of its high frequency, its clinical management is still controversial and even some of the most common clinical threats, such as intracystic infection, may represent an unmet challenge [3-7]. The clinical spectrum of infection in the kidney and liver cysts is protean: the disease ranges from mild abdominal discomfort, with a moderate increase of acute phase reactants, to severe life-threatening disease [2-7]. Diagnostic problems arise from the absence of a diagnostic gold standard in imaging and from the various, often non-specific clinical manifestations [4-7]. The need for long-term antibiotic therapy has to be weighed against the risk of side effects of prolonged treatment; the presence of kidney functional impairment may limit the use of many antibiotics [1-3].

The more vaguely defined "cystic kidneys", or multiple renal cysts, share with ADPKD the problem of definition and treatment of intracystic infections. The issue is also crucial in the case of acquired cystic disease of uremic patients [8,9].

Conventional imaging methods, including ultrasounds, CT (Computerized Tomography) and MR (Magnetic Resonance) scans, are valuable in discriminating between non-complicated and complicated cysts, but are often unable to clearly discriminate between bleeding and infection or, in particular in acquired cystic disease, neoplasia [3,5-9]. The concomitance of renal function impairment limits the potential of CT or MR scans, due to the relative or absolute contraindications for contrast media. Furthermore, the presence of several "complicated" cysts is common in severely enlarged liver or kidneys in ADPKD, and a complex structural derangement is usual in "acquired uremic cysts", often impairing the precise localization of the infectious process [1-3].

The use of scintigraphy with leucocytes labelled with indium or gallium has been reported as a promising diagnostic tool [10-12]. The limits of this technique are the lack of prompt availability, the high costs and the relatively poor spatial discrimination [13]. The first two limits are partly shared by Positron Emission Tomography (FDG-PET), able to identify metabolically active tissues, including infection, vasculitis and several types of neoplasia [14-18]. However, when associated with CT scanning, FDG-PET has good spatial discrimination, which may allow the guiding of percutaneous procedures or the study of the adjacent tissues [14-22]. The tracer, a glucose analogue, has strong avidity for most metabolically active tissues and is not toxic for the kidneys. These characteristics have led several Authors to consider FDG-PET/CT a technique of choice for the diagnostic work-up of fever of unknown origin [19-22].

Several recent case reports and two larger case series have suggested that FDG-PET is a very promising tool in the diagnosis of the infected kidney and liver cysts in ADPKD, probably superior to scintigraphy on account of its better spatial discrimination, particularly when combined with CT scanning [3,14-18]. In spite of the high potential interest, few studies have investigated the use of FDG-PET/CT in the diagnosis and follow-up of infected kidney cysts.

Using a search strategy on Medline in March 2010, including the key words "Postitron Emission tomography" and "kidney cysts", we were able to retrieve 5 case reports and small series, dealing with overall 7 patients. The widest experience was recently reported by a French group, describing promising results in 8 further cases studied with PET scan, within one of the largest recent retrospective analysis on cyst infections (41 episodes in 33 patients over a 10 year period) [3,14-18].

Here we report on 10 consecutive patients with suspected cystic infection (6 with ADPKD and 4 affected by multiple kidney cysts), in which the diagnosis was based and the clinical management was tailored upon the results of FDG-PET. This is, to our knowledge, the largest prospective series on the use of FDG-PET/CT in cystic kidney diseases, including both ADPKD and other cystic diseases of the kidney.

## Methods

### Study setting and patient selection

The present study regards a consecutive series of patients with suspected infection of a kidney or liver cyst, either in ADPKD or in kidney with multiple cysts. One case was referred within the work-up started for the presence of a non-symptomatic "complicated cyst".

Patients were either referred to the Nephrology Unit of San Luigi Gonzaga University Hospital, Orbassano, Turin, Italy (8 cases) or studied in cooperation with the caregivers of the Chair of Nephrology of the University of Turin (2 cases). The latter two cases were on renal replacement therapy (one on dialysis and one with a kidney graft).

The diagnosis of ADPKD was based upon the typical family history and shared criteria [1-3,23], consisting or, according to Pei et al [23], in the absence of family history, the presence of three or more renal cysts for individuals aged between 15 to 39, two or more cysts in each kidney for individuals aged 40 to 59 years, and four or more cysts in each kidney for individuals aged over 60 years. The definition of "isolated" kidney cysts was posed in the presence of a lower number of cysts as previously defined [23] and in the absence of any family history typical of ADPKD. Acquired cystic disease of the kidney was diagnosed in one patient with end stage kidney disease for chronic glomerulonephritis, who progressively developed multiple kidney cysts.

The diagnostic hypothesis of cystic infection was made on clinical and laboratory grounds. Systemic symptoms included fever, weight loss, malaise, loss of appetite; local symptoms included pain and abdominal discomfort, in particular if subacute or relapsing. Laboratory markers were the standard acute phase reactants (erythrocyte sedimentation rate, ESR, C-reactive protein, CRP, and fibrinogen); unexplained anaemia was an ancillary criterion. At difference with the reported retrospective criteria for a sure diagnosis of infection [3], the clinical criteria were broad and the prospective diagnosis was based upon the integration of the clinical follow-up, biochemical and imaging tests, aiming at identifying smouldering and early stages of infections, both in outpatients and in hospitalised patients [1-3,24].

Written informed consent was obtained from all the patients or their relatives for publication of study.

### Positron Emission Tomography

Positron Emission Tomography was performed in the same setting in all cases, both at the time of diagnosis and, in three cases testing positive at the first scan, during follow-up. In these cases a further scan was scheduled within one month after the start of antibiotic therapy and eventually repeated until complete remission. Patients were informed about the procedure and provided written informed consent. PET/CT studies were performed with the same Discovery ST scanner (General Electric Medical Systems, Waukesha, WI, USA). The patients were requested to refrain from food intake for at least six hours before scanning; at the time of tracer injection, all patients presented a blood glucose level under 160 mg/dL. Whole-body emission scans were acquired beginning 60 minutes after the intravenous injection of FDG (dose range: 222-370 MBq). The overall dose depended upon the weight of the patient, and as a rule depends upon the type of scanner. With our scanner the standard dose was 37 MBq for each 10 Kg of body weight.

The acquisition protocol started with a scout view (a two-dimensional CT projection of the patient), which was used to define the body axial extension (start and end position) over which to acquire the CT and PET data. When the scan range was defined, CT was performed (voltage 140 kV, tube current 60 mAs) from the proximal femur to the base of the skull. This scan lasted approximately 1 minute and was used for both anatomical localization and attenuation correction of the PET emission data. PET data on the whole-body distribution of the tracer were acquired in 3D mode from the pelvis to the neck (3 minutes per field of view [FOV]; 8-9 FOV). Coronal, sagittal and transverse data sets were reconstructed. The image reconstruction was performed as a 3D reconstruction algorithm FORE-Iterative, FOV: 50 cm, image matrix size: 128 × 128. All viewing of co-registered images was performed with dedicated software: Advantage 4.2 (GE Healthcare, Chalfont St. Giles, UK).

The SUV (Standardized Uptake Value) was calculated by drawing a region of interest (ROI) around the metabolically active lesion in PET images, i.e. we found the plane with the hottest voxel and then measured SUVmax for that plane using the formula: SUV = activity (MBq/mL) × body weight (g)/injected dose (MBq). We performed the same procedure for the two adjacent planes and then used the average of these measures for the analysis.

The identification of the lesions is based upon the intensity and on the extension, thus it's essentially qualitative. The SUV doesn't specifically contribute to the identification of the site, but may give some quantitative insights into the variation of the process over time. SUV was evaluated together with the extension for evaluation the clinical response. The localization of the infected site was based upon the fusion image. Furthermore, the inflammatory foci were confirmed by the acquisition of delayed images (at 30 minutes); in fact, infectious or inflammatory foci tend to increment the SUV over time while urinary activity decreases over time.

### Concordance analysis

Analysis of the concordance between CT and PET scans was performed by reviewing all the CT sequences acquired along with the PET scan.

Considering that contrast media are overall contraindicated in patients with reduced kidney function, the low dose CT without contrast media (included in our scan) was considered analogous to the CT scans which could have been performed for diagnostic purposes in our patients.

This analysis was employed to retrospectively evaluate the diagnostic yield of CT without contrast media (the most widely available imaging technique in almost every clinical setting).

The revision was performed by the same operator, an experienced radiologist (CG) unaware of the final PET results, after controlling for the diagnostic quality of all examinations.

In this critical revision, the results of the FDG-PET were reported by the Nuclear Medicine specialists, as they are routinely given. For the sake of the present analysis, the "isolated" images of the CT scan were reviewed by a radiologist, as previously specified.

The concordance between PET scan results and CRP levels (chosen as the most widely used routine marker of infection-inflammation) at diagnosis or during follow-up was also recorded.

The criteria employed are similar to those described by Sallée et coll, including enhanced wall thickening, perilesional inflammation, inflammatory fat stripes; an ancillary element at CT, not discriminating between bleeding and infection, was the presence of corpuscolated fluid in cyst [1-3,24].

## Results

### Clinical presentation

The main baseline clinical characteristics of the patients are reported in table 1.

**Table 1:** Main clinical data and comorbidities at diagnosis

Case	Sex	Age (yrs)	GFR (mL/min)	CKD stage	CRP mg/dL (normal < 0.5)	Fever (max)	Microbiological isolates	Cystic disease	Comorbidities-notes
1	F	60	On dialysis	V	Max 12 mg/dL	Max 38°C relapsing; often after dialysis	None	ADPKD, large liver and kidney cysts	Left nephrectomy for transplant wait listing 3 years previously
2	M	77	25	IV	Max 10 mg/dL	Max 38°C	None	Multiple kidney cysts, hypertensive nephrosclerosis	Severe cardiovascular disease, right nephrectomy for neoplasia 15 years previously
3	F	68	101	I	Max 2.4 mg/dL	Low-grade	E Coli (urine)	ADPKD, liver and kidney cysts	Previous cerebellar haemorrhage
4	M	83	18	IV	Max 5.5 mg/dL	Low-grade	E Coli (urine)	ADPKD, large liver and kidney cysts	Severe cardiovascular disease; blindness
5	F	55	40	III	Max 1.2 mg/dL	No	Frequent UTI (E Coli in the last episodes)	ADPKD, large liver and kidney cysts	Anxiety-depression, Child A cirrhosis; breast neoplasia
6	F	81	35 at hospitalization; 65 after therapy	IV - II	Max 36 mg/dL	Max 39.5°C	E Coli (urine)	Two large kidney cysts	Previous TIA, moderate hypertension
7	F	65	35	III	Max 0.8 mg/dL	Occasional low-grade	none	ADPKD, liver and kidney cysts	Anxiety-depression
8	M	71	70	II	Max 22 mg/dL	Max 40°C	Proteus Mirabilis (urine) 2 weeks before hospitalization	Multiple kidney cysts	GFR decrease to 35 mL/min on therapy with aminoglycosides
9	F	63	55	III	Max 1.0 mg/dL	No	Frequent UTI (E Coli, last one one month previously)	Acquired cystic disease	Kidney transplant recipient
10	F	68	24	IV	Max 13.7 mg/dL	Max 38°C	Frequent UTI; last episode 2 weeks previously (self treated)	ADPKD, large kidney cysts, few liver cysts	Hypertension, hyperparathyroidism

Legend: F: female, M: male; GFR: glomerular filtration rate (Cockcroft formula or 24 hours urine collection); CRP: C-Reactive Protein; ADPKD: Autosomic dominant polycystic kidney disease. UTI: Urinary tract infections

The study group was non homogeneous for CKD stage and kidney disease. Six patients were affected by ADPKD, three had multiple renal cysts and one patient, a kidney transplant recipient (case 10), was affected by acquired cystic disease. One patient (case 1) was on haemodialysis and 4 patients (cases 2, 4, 7, 10) had marked reduction of renal function, limiting the possibility of using conventional contrast media (table 1).

In all but one patients the clinical picture was characterised either by the presence of acute or subacute inflammatory signs or fever and abdominal discomfort. In a single case (case 9, a kidney transplant patient) FDG-PET/CT was performed in the diagnostic-work-up for positive urinary cytology in acquired cystic disease, to rule out a smouldering infection before biopsy of the largest cysts or nephrectomy.

All patients had undergone at least one imaging study (ultrasounds in all, CT scan in 8).

**Figure 1:**
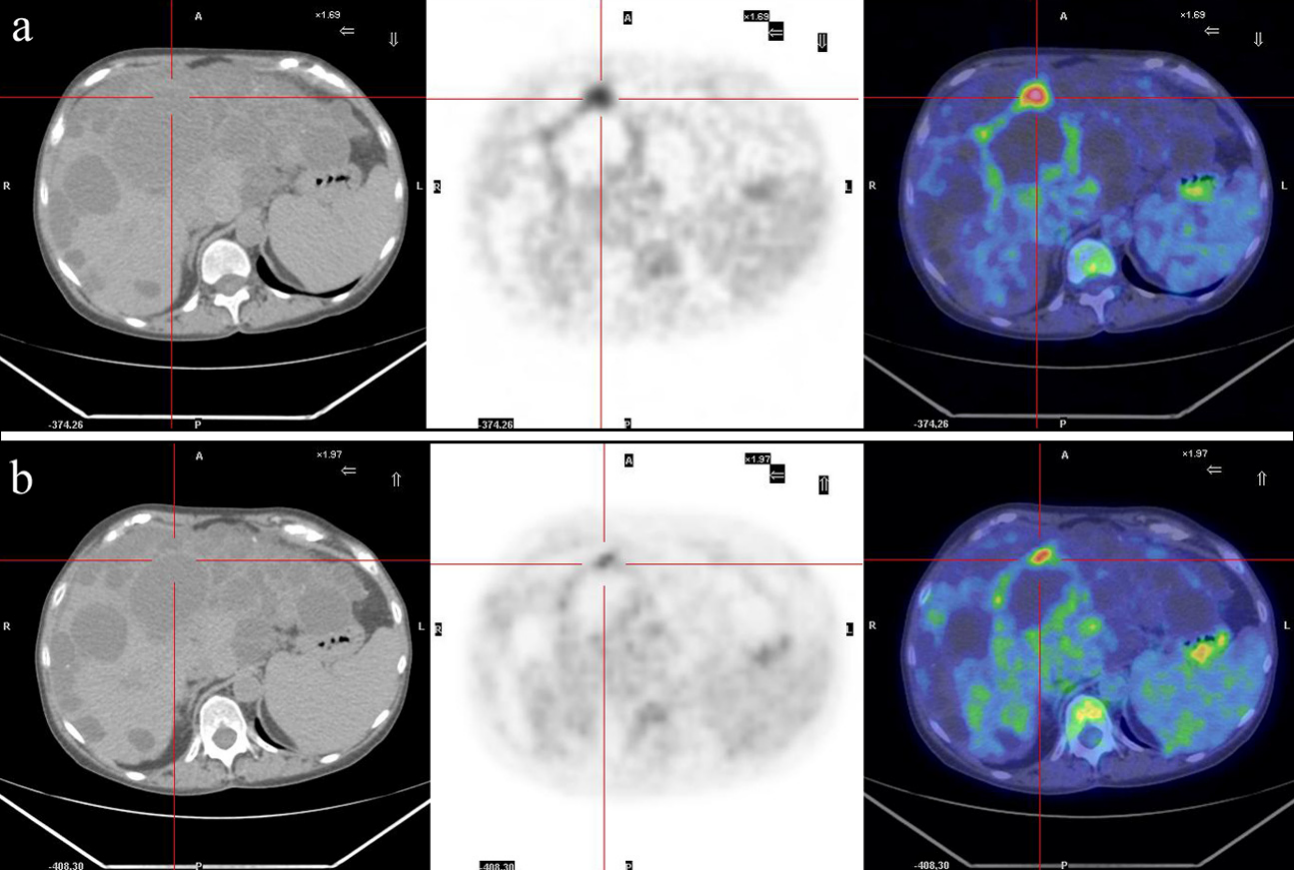
**FDG-PET scan: intracystic infection in a large liver cyst in a ADPKD patient, before and after antibiotic therapy**.

**Figure 2:**
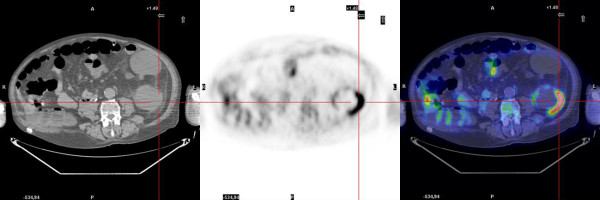
**FDG-PET scan: intracystic kidney infection in a CKD patient with multiple cysts**.

**Figure 3:**
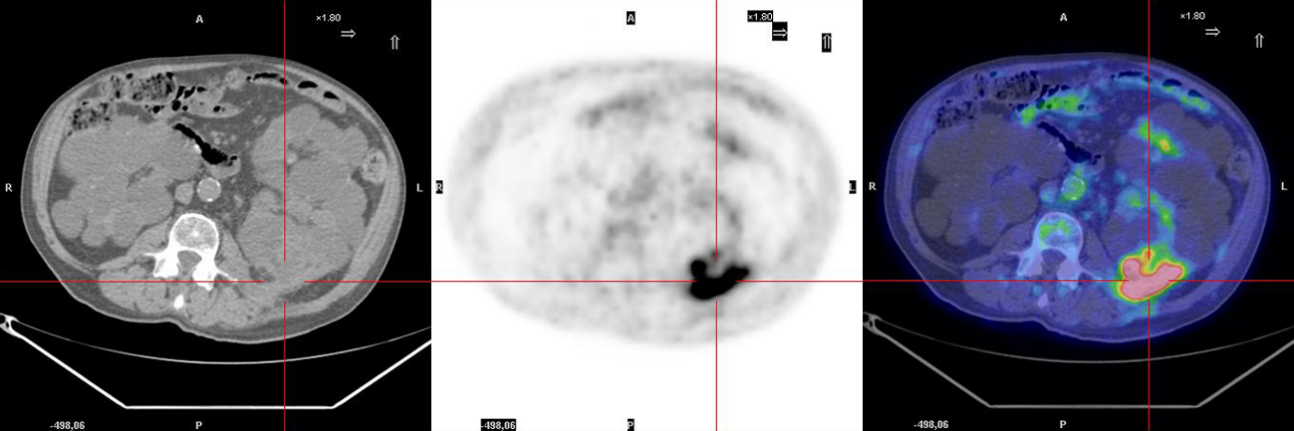
**FDG-PET scan: intracystic kidney infection in ADPKD**.

FDG-PET revealed the presence of metabolic activity suggestive of infection in 6 patients. The settings were a liver cyst in one case with ADPKD (case 1, Figure 1) and a kidney cyst in 5 cases, 3 affected by ADPKD and 2 with multiple cysts (cases 2-6, Figures 2, 3). No metabolic activity was recorded in 4 cases (cases 7-10). In case 10, FDG-PET identified the presence of metabolic activity in a lymph node (about 3 cm of maximum size), already disclosed by a CT scan one year previously and apparently stable at the CT scan preformed during the studied episode. Hence, taking into account the high metabolic activity at the PET scan, the patient underwent an ultrasound-guided agobiopsy, revealing a mesenchymal neoplasia with low replicating potential. The patient is presently on oncologic follow-up (table 2).

**Table 2:** Main indications for FDG-PET

Case	Sex	Age	Main symptoms and indications for FDG-PET	Other imaging tests performed
1	F	60	Relapsing fever, with abdominal discomfort, not responsive to short-term antibiotic therapy; high CRP.	US; CT scan with contrast media (3 complicated liver cysts)
2	M	77	Relapsing high fever, increased serum creatinine and CRP.	US; CT scan without contrast media
3*	F	68	Low-grade fever, relapsing after short cycles of antibiotics; vague abdominal discomfort, malaise, weight loss.	US
4	M	83	Low-grade fever, abdominal pain; anaemia; increased serum creatinine and CRP, malaise, weight loss.	US; CT scan without contrast media
5	F	55	Worsening of the usual vague abdominal complaints; mild increase in CRP.	US; CT scan without contrast media
6	F	65	Occasional low-grade fever, abdominal discomfort and pain enhanced by prolonged standing; malaise, weight loss.	US
7	F	81	High grade fever; flank pain; symptoms of lower UTI	US; CT scan without contrast media
8	M	71	Referred after hospitalization in a different setting for fever and upper UTI; FDG-PET after 1 month of antibiotics to assess the presence of residual disease or indication for cyst drainage.	US; CT scan with and without contrast media
9	F	63	Differential diagnosis between infectious and neoplastic complication in acquired cystic disease.	US; CT scan without contrast media
10	F	68	Fever at hospitalization with abdominal pain and macrohematuria; lower urinary tract infection.	US; CT scan without contrast media

Legend: F: female, M: male; US: ultrasounds: computed tomography; MR: Nuclear magnetic resonance CRP: C-Reactive Protein;

UTI: urinary tract infection. *The main clinical features of Case 3 were published on NDT Plus (25).

### Therapy and follow-up

Table 3 summarises the main imaging findings at diagnosis and during follow-up and the therapeutic choices performed integrating the clinical features and the imaging data.

**Table 3:** Main findings at FDG-PET

Case	PET n; interval	Main finding at FDG-PET	Main finding at CT scan, concomitant to PET	SUV	Therapy	Outcome
1	1	High pathological FDG uptake in two liver cysts in the IV segment	Several mainly hypodense liver cysts, Right kidney is enlarged with multiple, hypo and hyperdense cysts. Stable at follow up studies	7.6	Combined therapy: amikacine and ceftriaxone for one month; ceftriaxone for 2 months	No relapse; kidney graft
	2 (6 weeks)	Significant reduction in extension of pathological FDG uptake; non significant reduction in SUV	No substantial change	7.0		
2*	1	High pathological FDG uptake in the large exophytic cyst of the left kidney, no uptake in the lower polar cyst	Along the posterior margin of the middle left kidney, hyperdense renal cyst with contiguous thickening of the perirenal fat and dense pericystic band. Other cysts with thickened walls in lower pole.	13.3	Combined therapy: amikacine and ceftriaxone for 2 weeks, ceftriaxone for 1 month	Died from acute myocardial infarction 1 year later
3	1	High pathological FDG uptake in a cyst in the left kidney	both kidneys are slightly enlarged. Multiple hypo and hyperdense cysts at both sides. Subtle bands in the perirenal right fat	3.8	Combined therapy: amikacine and ceftriaxone for 1 week, ceftriaxone for 6 weeks	No relapse, normal kidney function
	2 (5 weeks)	Significant decrease of pathological FDG uptake	The right perirenal bands are attenuated	3.2		
	3 (4 weeks)	No pathological FDG uptake	Unchanged	-		
4	1	High pathological FDG uptake in left polycystic kidney	Both kidneys are enlarged with multiple, mainly hypodense cysts, In the middle of the right a non-homogeneous cyst, with ill defined margins, with thickened perirenal fat tissue	16.0	Ceftriaxone for 1 month	No relapse, stable kidney function
5	1	Pathological FDG uptake in left kidney cysts	Multiple liver cysts, hyper and hypodense. Both kidneys are enlarged with multiple, hypo and hyperdense cysts.	2	Ceftriaxone for 3 weeks, followed by oral amoxicillin-clavulanate for 1 month; therapy stopped at the time of the 3^rd ^PET (minimal uptake)	No relapse, stable kidney function
	2 (3 weeks)	Reduction of pathological FDG uptake	Unchanged	1.8		
	3 (4 weeks)	Further reduction of pathological FDG uptake	Unchanged	1.6		
	4 (4 weeks)	No pathological FDG uptake	Unchanged	-		
6	1	High pathological FDG uptake in the large (9 cm) kidney cyst	Two large kidney cysts, the largest one with non homogeneous fluid and thickened walls. Thickened perirenal fat tissue.	7.5	Amikacine for 2 weeks, initially with ertapenem, and later with ceftriaxone (leukopenia and anemia) overall 9 weeks of i.v. therapy	No relapse, improved kidney function
	2 (3 weeks)	Initial reduction of FDG uptake	Unchanged	7		
	3 (5 weeks)	Almost complete resolution of the FDG uptake	Unchanged cystic appearance; resolution of the pericystic infiltrate	3		
7	1	No pathological FDG uptake	Few complicated cysts in both kidneys	-	No antibiotic therapy	Well, no infectious complication
8	1	No pathological FDG uptake	In the upper pole of right kidney a hypodense cyst with ill defined margins, near to a thickened Gerota fascia	-	Before referral: amikacine, chinolones, cefalosporins. After referral: 3 weeks of cefalosporins followed by oral amoxicillin clavulanate	Well, one episode of lower UTI after discontinuation of therapy
9	1	No pathological FDG uptake in left kidney cysts	Acquired cystic disease with several hypo and hyperdense cysts	-	No therapy	Scheduled for biopsy of the main "complicated" cysts
10	1	No pathological FDG uptake in bilateral polycystic kidneys. Collaterally detected high pathological FDG uptake in peripancreatic lymph node	Large polycystic kidneys with multiple hyper and hypodense cysts present in both kidneys. Large peripancreatic lymph-node (3 cm) stable as compared to a previous CT scan	-	Cephaoloporines for urinary tract infection ("cysto-pyelitis"), for 3 weeks; agobiopsy: mesenchymal neoplasia with low prolipheration index	On further oncologic diagnostic work-up

Legend: SUV: standardized uptake value; PET: Positron emission tomography; FDG: fluoro desoxy glucose;

* one PET only, for clinical and logistical reasons in severely compromised vasculopathic patients.

Four of the 6 patients testing positive at the first scan underwent another 8 FDG-PET/CT scans; of note, two cases presented a smouldering picture, in which the FDG-PET/CT scan was the main tool for tailoring the duration of the antibiotic therapy (table 3). In these cases, the metabolic activity resolved slowly with long-term antibiotic therapy. In two cases, with severely compromised clinical conditions, only one imaging study was feasible and long term antibiotic therapy (4-6 weeks) was empirically decided on the basis of the first FDG-PET/CT scan.

The therapeutic strategy was based on the clinical picture and CKD stage. In the patients with GFR > 50 mL/min and in the patient already on dialysis, aminoglycosides were used, at least for the first week of therapy, on the basis of their pharmacological properties, allowing rapid sterilization of blood and urine. In these cases, aminoglycosides were combined with and followed by either third-generation cefalosporins (ceftriaxone) or by carbapenemic antibiotics (ertapemen) until resolution of the metabolic activity. In the cases with lower GFR, cefalosporins or carbapenemics alone were employed. All patients testing positive at FDG-PET/CT underwent at least one month of intravenous or intramuscular therapy (table 3).

None of the positive patients with at least 6 months of follow-up relapsed after the discontinuation of therapy, and no patient testing negative at FDG-PET/CT developed a clinical picture suggestive of intracystic infection (table 3).

The therapeutic decision in case 7 (further antibiotic therapy, in spite of negative FDG-PET/CT) was based on the presence of a mild residual elevation of CRP (1-1.5 mg/dL, normal values < 0.5 mg/dL), according to the protocols of the infectivologists, suggesting to discontinue therapy after one week of normal CRP values. In this case the results of FDG-PET/CT were an argument for avoiding the option of cystic puncture, strongly suggested by the interventional radiologists.

### Concordance analysis

At diagnosis, all but one patient (case 9) displayed a mild to severe increase of acute phase reactants. However, the level of CRP, usually chosen as the main marker of infection, was only mildly elevated in 3 cases (2 testing positive at FDG-PET/CT scan and 1 testing negative). The CRP level did not correlate with the presence of fever or the severity of the clinical symptoms, suggesting "background noise" of the presence of multiple comorbidities in a relatively old population with CKD (table 1 table 2). Normalization of CRP was observed in the positive patients within 3 weeks of antibiotic therapy; the normalization of CRP preceded the normalization of PET scans in the three patients who underwent further FDG-PET/CT scans.

Revision of the CT images recorded during the PET scan allowed identification of the "complicated cysts" in most of the cases, but not the precise localization of active infection. The main clue for infection was indirect: the presence of perinephric fat inflammation. Likewise, the CT scans were less sensible to identify the intracystic changes over time (table 3).

## Discussion

Diagnosis and treatment of intracystic infections is still a challenge in patients with ADPKD and with other types of multiple kidney cysts [3-7,24]. There are two main points in this challenge: diagnosis of infection and identification of the infected cyst, and definition of type and duration of therapy.

The described cases shared the first diagnostic challenge, i.e. identification of the presence and site of the infectious foci (table 1; table 2). In contrast with acute pyelonephritis, usually diagnosed in the presence of high fever and severe systemic complaints, the diagnosis of intracystic infection may not be evident, with non-specific complaints and a smouldering clinical picture [1-7,24]. The wide clinical spectrum of presentations is represented in our series, ranging from severe infection to mild oligosymptomatic disease, also including cases referred late after several attempts at empirical antibiotic treatment (table 1 table 2 table 3).

In four cases, the clinical picture was that of a clinically important infection, with increased inflammatory markers, fever and abdominal or flank pain; in three of them, FDG-PET/CT identified metabolic activity in liver (case 1) or kidney cysts (cases 2, 4). In the fourth case (case 10) FDG-PET/CT disclosed metabolic activity in a large peripancreatic lymph node; this led to diagnosis of mesenchimal neoplasia and offered an alternative diagnosis for the non specific abdominal pain, while the urinary tract infection was successfully managed with a relatively short antibiotic course.

Two patients were referred late to our Unit after long-term antibiotic therapy (cases 3 and 7); FDG-PET/CT was positive in one (case 3) and negative in the second patient (case 7), who presented a CT picture suggestive of slowly resolving perinephric soft tissue infiltration contiguous to two large kidney cysts.

In three cases, the clinical picture was mild and the increase of the inflammatory markers was minor; once more, as extensively described in the literature, neither the clinical picture nor the CT scan allowed discriminating among these non specific presentations (table 1 table 2 table 3).

One of the three tested positive at FDG-PET/CT and was treated by long-term antibiotic therapy, with improvement of the symptoms and normalization of the inflammatory markers.

Regarding the second challenge, i.e. therapy, the lack of full concordance between the clinical presentation, biochemical data, conventional imaging techniques and FDG-PET/CT underlined the importance of refining the diagnostic procedure in order to guide the therapy (table 1 table 2 table 3). Our therapeutic policy is mainly based on medical management; in this series, none of our patients underwent cyst aspiration, and long-term antibiotic therapy only was performed in positive cases.

In keeping with the literature, in patients with good renal function or on dialysis we associated an aminoglycoside with an antibiotic with good diffusion profile (cefalosporins or carbapenemics), using the latter only in severe CKD [1-3,7,14-18]. At difference with other series, the choice of fluoroquinolones was limited by the presence of a 30-40% rate of resistance in our setting, and by the fear of nephrotoxicity, in particular in late CKD stages [3,24].

In keeping with the literature, reporting lack of identification of the causative agent in about one fourth of cases, in patients hospitalized, with severe infection, also in our series the identification of the infectious agent was possible in only about half of the cases. Furthermore, while the identification of the putative agent is important for tailoring therapy, it did not discriminate between intracystic and other types of urinary tract infections (table 1 table 2 table 3).

Whenever possible, the duration of therapy was tailored on further FDG-PET/CT results; however, a second scan was not feasible in two elderly patients because of severe cardiovascular problems and compromised clinical conditions (table 2 table 3).

This point is however a crucial one, as the relationship between the persistence of the altered image and of infections is not proven; our choice to consider them synonymous should thus be considered at present as a working hypothesis only.

It is noteworthy that the therapeutic choices were affected by the FDG-PET/CT results in both negative and positive cases; for instance, they supported the decision to avoid cyst puncture in case 8, in contrast to the initial opinion of the interventional radiologists, and led to the incidental discovery of an abdominal lymph node in case 10, thus modulating the overall clinical approach.

The timing of the response is a crucial problem. The response to chemotherapy or radiotherapy, for which there is presently a large amount of data, is intrinsically different from the response to infection. Intracystic infections may moreover be different as the diffusion of antibiotics may be altered and the inflammation may persist longer.

In evaluating the response to chemotherapy or radiotherapy, 3-12 weeks are needed according to the therapy (chemotherapy or radiotherapy). The best timing for evaluating the response is not jet assessed in cystic diseases of the kidney, as a relatively small number of cases has so far been reported. However, the variation in the extension of FDG uptake and in the SUV may help tailoring therapy, confirming the initial efficacy of the chosen treatment; thus our policy during follow-up was tailored upon, even if not based upon, FDG-PET/CT results.

None of our positive cases relapsed after discontinuation of therapy and none of the negative cases developed a clinically active infection, supporting a good clinical-imaging correspondence. The possibility to perform a whole body scan may allow disclosing other infectious-inflammatory or neoplastic foci, as it occurred in case 10.

Our study has several limitations, partly shared by non-controlled observational case series in new clinical fields. It reports on a small cohort of inhomogeneous cases and is based on a single-centre experience in which the presence of skilled radiologists and nuclear medicine experts allows tailoring of patient management based on imaging data. As underlined by several Authors the diagnosis of intracystic infection is not clear-cut and the criteria are not jet univocal [1-3,7,24]. Thus, in a setting where cystic puncture is not routinely performed, the diagnosis of intracystic infection cannot definitively be proved. However, the concordance with the follow-up data (no relapse in treated cases; no development of clinical infection in negative cases) is a strong argument supporting relying on PET results, in a clinical context in which, due also to age and comorbidity, invasive procedures are preferentially avoided (table 1).

The low availability and high cost of FDG-PET/CT are probably the main limitations to its wider use. These limits are shared also by the two alternative techniques of scintigraphy with leucocytes labelled with indium or gallium; thus the experience is relatively limited in spite of a longer history of availability of these techniques [10-12]. The relatively poor spatial discrimination, the high radiation activity and the time consuming and complex preparation needed for these techniques may support a wider use of FDG-PET/CT scan, whose costs are comparable in several settings, including ours [13].

However, since less than 30 cases (including ours) have been reported in the literature, the drawbacks of the technique may not yet be clear [24].

The strengths of our study are the fact that it's the first relatively large prospective study, and the first one to report on the use of FDG-PET/CT for suspected infection not only in ADPKD but also in other cystic diseases of the kidney.

Both the limits and strengths suggest the need of further studies to define a favourable cost-benefit profile of the procedure and precise indications for its application.

## Conclusions

FDG-PET/CT is a very promising tool for the diagnosis and follow-up of infected cysts in ADPKD or in other cystic diseases of the kidney. Further studies are needed to better assess the cost-benefit ratio in order to guide the systematic use of this technique in the complications of ADPKD or other types of cystic kidney diseases.

## Competing interests

The authors declare that they have no competing interests.

## Authors' contributions

PG conceived the study, participated in its design and coordination of the clinical management of the patients and drafted the manuscript; CV was encharged of the clinical management of the patients; DM participated to the clinical management, and made the bibliographic systematic search; AV coordinated the imaging studies and participated to the study design; PE was encharged of the review of the PET analyses; AD participated in the critical review of the manuscript and in the imaging analysis; PD participated in the imaging analysis and in the critical review of the images; CG participated in the study design, in the manuscript draft and reviewed CT scans. All authors read and approved the final manuscript.

The authors also declare that they have made a substantial contribution to the information or material submitted for publication the manuscript has been read and approved by all authors in this version the manuscript or portions thereof are not under simultaneous consideration by any other journal or electronic publication and have not been previously published.

## Pre-publication history

The pre-publication history for this paper can be accessed here:

http://www.biomedcentral.com/1471-2369/12/48/prepub
